# Desnutrição e Caquexia na Insuficiência Cardíaca Descompensada e Cardiomiopatia Chagásica: Ocorrência e Associação com Desfechos Hospitalares

**DOI:** 10.36660/abc.20200644

**Published:** 2021-11-17

**Authors:** Larissa Candido Alves Tavares, Silvia Helena Gelás Lage, Edimar Alcides Bocchi, Victor Sarli Issa

**Affiliations:** 1 Universidade de São Paulo Instituto do Coração São Paulo SP Brasil Universidade de São Paulo Instituto do Coração - Serviço de Nutrição e Dietética, São Paulo , SP – Brasil; 2 Universidade de São Paulo Instituto do Coração São Paulo SP Brasil Universidade de São Paulo Instituto do Coração - Unidade de Terapia Intensiva Clínica, São Paulo , SP – Brasil; 3 Universidade de São Paulo Instituto do Coração São Paulo SP Brasil Universidade de São Paulo Instituto do Coração - Insuficiência Cardíaca, São Paulo , SP – Brasil

**Keywords:** Cardiomiopatia Chagásica, Insuficiência Cardíaca, Desnutrição, Caquexia, Avaliação Nutricional

## Abstract

**Fundamento:**

Problemas nutricionais são comuns em pacientes com insuficiência cardíaca (IC) e estão associados a um prognóstico ruim. É relevante mencionar que algumas populações de pacientes, como os com Doença de Chagas, são normalmente excluídas da maioria das análises.

**Objetivo:**

Buscamos analisar a ocorrência de desnutrição e caquexia em pacientes com Doença de Chagas durante episódios de IC descompensada (ICD) em comparação a outras etiologias, e investigar a influência desses achados em desfechos hospitalares.

**Método:**

Realizamos um estudo de série de casos consecutivos com pacientes hospitalizados com ICD. Os pacientes foram submetidos à Avaliação Nutricional Subjetiva Global (ASG), além de medidas antropométricas e laboratoriais, e foram avaliados para a ocorrência de caquexia, baixa massa muscular e força. Estudamos a ocorrência de morte e transplante cardíaco de urgência durante a internação.

**Resultados:**

Ao todo, 131 pacientes foram analisados e 42 (32,1%) tinham Doença de Chagas. Pacientes com Doença de Chagas apresentavam índice de massa corporal (IMC) menor (22,4 kg/m2 [19,9-25,3] vs. 23,6 kg/m2 [20,8-27,3], p=0,03), maior frequência de desnutrição (76,2% vs 55,1%, p=0,015) e mais ocorrências de morte ou transplante (83,3% vs. 41,6%, p<0,001). Observamos que, dentre os pacientes com etiologia da Doença de Chagas, a ocorrência de morte ou transplante cardíaco esteve associada com desnutrição (3 [42,9%] pacientes com alta hospitalar vs. 29 [82,9%] pacientes que morreram ou receberam transplante cardíaco, P=0,043).

**Conclusões:**

Ao todo, nossos resultados indicam que pacientes com Doença de Chagas internados com ICD costumam apresentar problemas nutricionais, principalmente desnutrição. É importante mencionar que este achado esteve associado à ocorrência de morte e transplante cardíaco durante a internação.

## Introdução

Problemas nutricionais são uma das principais manifestações clínicas em pacientes com insuficiência cardíaca (IC), e são resultado de desordens sistêmicas envolvendo vias metabólicas, endócrinas e inflamatórias, assim como disfunção de órgãos. A ocorrência de distúrbios nutricionais no cenário da IC crônica esteve consistentemente associada à redução na qualidade de vida e sobrevivência dos pacientes.^1 - 5^

As disfunções nutricionais podem se manifestar de várias formas clínicas, o que se reflete em uma terminologia extensa e heterogênea.^1 - 5^ Mais recentemente, uma padronização de termos foi proposta^6 , 7^ e sugeriu que a *desnutrição* pode ser definida como uma condição na qual a energia e as reservas de proteína do organismo estão debilitadas; enquanto isso, a caquexia foi caracterizada pela intensa perda de gordura e massa muscular, acompanhada do aumento da inflamação e do catabolismo de proteínas devido a doenças crônicas.

Estima-se que a frequência de caquexia e desnutrição associada à IC mude de acordo com a população estudada e os critérios diagnósticos. Além disso, a retenção de água frequente nesta população afeta medidas básicas para a avaliação nutricional, como peso e Índice de Massa Corpórea (IMC), o que se torna um desafio na prática clínica. A estimativa é de que em torno de 15% dos pacientes com IC crônica apresentem caquexia, e que até 50% dos pacientes podem apresentar sinais de desnutrição;^2^ além disso, uma prevalência de 19,5% de atrofia muscular foi relatada.^3^ Porém, a maioria dos dados deriva de pacientes com IC crônica, e poucos estudos foram sistematicamente reportados considerando a avaliação nutricional de pacientes durante episódios de descompensação aguda. Em um dos poucos estudos sobre IC descompensada (ICD), uma prevalência de 41,9% de desnutrição moderada foi relatada, enquanto 7,4% apresentou desnutrição grave, conforme a Avaliação Nutricional Subjetiva Global (ASG).^4^ Embora a ASG tenha sido descrita 20 anos atrás,^8^ o método demonstrou boa precisão diagnóstica quando realizado por observadores treinados; além disso, é simples, econômico e não-invasivo.^9^ A ASG avalia o diagnóstico do status nutricional, diferentemente de outras ferramentas, como o Índice de Prognóstico Nutricional (IPN) e o Controle do Estado Nutricional (CONUT). Embora sejam frequentemente usados na prática clínica, são basicamente ferramentas de triagem e não se aplicam ao diagnóstico de desnutrição.^10^

Dados nutricionais são ainda mais escassos em outros cenários clínicos, como em populações de pacientes normalmente excluídas de ensaios clínicos e estudos prospectivos, referidas como estando em condições negligenciadas,^11^ como é o caso da Doença de Chagas.^12^ Apesar de evidências mostrarem que pacientes com Doença de Chagas têm pior prognóstico em casos de IC crônica^13^ e descompensada,^14^ em comparação a outras etiologias, o quanto as características nutricionais de pacientes com Doença de Chagas se diferenciam e seu impacto no prognóstico ainda são muito pouco estudados.

Assim, nossa hipótese é a de que os distúrbios nutricionais sejam comuns em pacientes com ICD e tenham uma influência no prognóstico; também consideramos que as características nutricionais dos pacientes podem se diferenciar em termos de intensidade, apresentação clínica e implicação prognóstica de acordo com a etiologia da IC.

O objetivo deste estudo foi avaliar a ocorrência de desnutrição e caquexia em pacientes com ICD e observar características clínicas e nutricionais e sua influência em desfechos hospitalares de acordo com a presença da etiologia da Doença de Chagas.

## Pacientes e Métodos

### Desenho do Estudo

Realizamos um estudo de série de casos consecutivos com pacientes internados e diagnosticados com ICD no Instituto do Coração (InCor), Hospital das Clínicas, Faculdade de Medicina da Universidade de São Paulo, um hospital acadêmico terciário especializado em doenças cardíacas. O protocolo do estudo foi aprovado pelo Comitê de Ética em Pesquisa do Hospital das Clínicas, Faculdade de Medicina da Universidade de São Paulo, e todos os pacientes assinaram o termo de consentimento. Os pacientes foram acompanhados até a alta hospitalar.

Pacientes com 18 anos ou mais, internados com ICD e com disfunção sistólica ventricular esquerda foram considerados elegíveis para o estudo. A fração de ejeção ventricular esquerda (FEVE) menor do que 50%, medida pelo ecocardiograma transtorácico foi considerada como indicativo de disfunção sistólica. Os testes foram realizados até doze meses antes de o episódio de descompensação ser considerado válido. A primeira inclusão aconteceu em fevereiro de 2016, e a última foi em abril de 2018.

A identificação de casos se baseou nos registros médicos que reportavam o diagnóstico de ICD. Para ser incluído no estudo, o paciente precisava estar de acordo com os critérios de Framingham para o diagnóstico de IC.^15^ Os seguintes critérios foram considerados como indicativos da descompensação: presença de qualquer novo sintoma ou piora dos sintomas atuais (falta de ar, ortopneia, edema periférico, ascite), combinada com qualquer outro sinal de congestão ou hipoperfusão (taquicardia, hipotensão, dispneia, taquipneia, edema de membros inferiores, crepitações pulmonares, efusão pleural, ascite, hepatomegalia, maior pressão venosa central e presença de refluxo hepatojugular). O diagnóstico da etiologia da Doença de Chagas se baseou na sorologia positiva para a condição, junto com a apresentação clínica típica e a exclusão de outras etiologias.

Os critérios de exclusão foram: presença de doença cardíaca congênita; cardiomiopatia restritiva; teste positivo de HIV; alcoolismo ativo; doença pulmonar obstrutiva crônica (DPOC) limitante; presença de megacólon chagásico e/ou megaesôfago; uso contínuo de corticosteroides ou imunossupressores nos últimos 3 meses; neoplasia maligna; embolia pulmonar nos últimos 6 meses; grandes cirurgias ou infecções graves nos últimos 30 dias; doenças valvulares primárias como causa da IC; limitações físicas que impossibilitaram as medidas antropométricas de forma minimamente adequada; impossibilidade de realizar a anamnese clínica e nutricional com o paciente, parente ou acompanhante.

A partir de julho de 2017, o tempo de internação hospitalar maior que 7 dias antes da inclusão no estudo foi adicionado como critério de exclusão.

## Resultados estudados

Pacientes foram acompanhados desde a internação até a alta, em casos de morte ou transplante cardíaco de urgência.

### Variáveis clínicas e nutricionais

As variáveis clínicas da amostra foram obtidas a partir da revisão da evolução médica computada em registros médicos eletrônicos e entrevistas com pacientes e/ou parentes.

Valores de hemoglobina (Hb), glicose, hemoglobina glicada (HbA1c), colesterol total, colesterol LDL, colesterol HDL, triglicérides (TG), albumina, contagem total de linfócitos (CTL), proteína C-reativa (PCR) foram coletados durante a internação. Para o peptídeo natriurético tipo-b (BNP), consideramos qualquer mensuração feita até 6 meses antes da internação como válida.

As variáveis não-bioquímicas relacionadas ao status nutricional foram obtidas a partir de uma entrevista com o paciente e/ou acompanhante, e a avaliação das medidas antropométricas foi realizada por um único nutricionista clínico. As medidas de altura, peso atual e usual foram referidos pelo paciente. A altura e o peso dos pacientes que conseguiam andar também foram verificados com uma balança digital com estadiômetro acoplado, com capacidade para até 150kg e 190cm (Filizola®). Em casos em que os indivíduos tinham edema na hora da avaliação, o peso atual desconsiderando o edema foi reportado pelo paciente, e o indivíduo não foi pesado na balança.

Pacientes que não podiam andar por causa da medicação, mas conseguiam ficar em pé, foram pesados com uma balança digital portátil (EKS 8873 DOMUS Plataforma ABS®). A altura desses mesmos pacientes foi medida com um estadiômetro portátil (Wood Portátil Compact®). Indivíduos que não souberam reportar peso e altura e aqueles acamados, que não podiam ser pesados, tiveram suas alturas e pesos estimados por fórmulas preditivas. Essas fórmulas consideram altura do joelho, idade, circunferência do braço (CB) e etnia.^16 - 18^

A CB foi medida com uma fita métrica no ponto médio do braço (entre o acrômio e o olecrano), com o braço esticado livremente ao longo do corpo. A dobra cutânea tricipital (DCT) foi medida com um adipômetro (Sahean®). A dobra foi pinçada no ponto médio do braço (entre o acrômio e o olecrano), com o braço esticado livremente ao longo do corpo. A medida foi repetida três vezes, e a média das medidas foi utilizada para análise. A força de preensão palmar (FPP), considerada como medida de força isométrica, medindo o aperto de mãos no membro superior dominante, foi medida com um dinamômetro digital (MG-4800®). O teste foi realizado com o indivíduo sentado, ou com a cabeça elevada a pelo menos 30º quando o paciente tinha um balão intra-aórtico, e o braço formando um ângulo de 90º com o apoio do cotovelo. Três medidas de força máxima foram realizadas com intervalos de 10 segundos entre elas, e a média dos três valores foi considerada.

### Diagnóstico e categorização

O IMC foi categorizado de acordo com os critérios da Organização Mundial da Saúde (OMS)^19^ para adultos, e considerando os critérios da Organização Pan-Americana de Saúde (OPAs)^20^ para idosos com mais de sessenta anos. Para diagnosticar a desnutrição, utilizamos a ASG.^8^ A aplicação de todos os questionários e exame físico foi realizada por um único nutricionista clínico. A identificação de indivíduos com baixa massa muscular foi realizada ao calcular a área muscular do braço (AMB), obtida por meio da CB e da DCT, usando a seguinte fórmula:


$$\text{ AMB }\left({cm}^{2}\right)={\left[CB(cm)-\left(DCT(cm)\times \prod ÷10\right)\right]}^{2}/4\Pi$$

Indivíduos com baixa massa muscular foram aqueles abaixo do 10º percentil quando comparados à população de referência. Os valores comparativos de acordo com as porcentagens da população de referência foram obtidos a partir da distribuição apresentada por Frisancho^21^ para adultos, e da distribuição apresentada por Burr e Phillips^22^ para idosos.

O diagnóstico de força muscular reduzida foi realizado por meio da FPP. Para fins de análise, indivíduos com baixa força muscular foram os que apresentaram valores iguais ou menores do que aqueles considerados por Mathiowetz *et al* .^23^ em uma população saudável, de acordo com gênero e faixa etária.

O diagnóstico de caquexia cardíaca foi realizado com base nos critérios propostos por Evans *et al* .^24^ Esta definição envolve a presença de um diagnóstico de doença crônica (neste estudo, representada pela presença de IC), associado à perda de peso de 5% em relação ao peso usual em um período máximo de 12 meses, ou IMC menor que 20kg/m^2^ , acompanhado de pelo menos três dos critérios seguintes: fadiga, anorexia, baixa força muscular, baixa massa muscular e anormalidades bioquímicas, como marcadores inflamatórios aumentados, anemia e albumina sérica baixa.

A gravidade da IC foi estimada neste estudo ao observar a ocorrência de níveis mais altos de BNP circulante e valores menores de FEVE, conforme demonstrado na literatura.^25 , 26^

### Análise estatística

O teste de Kolmogorov-Smirnov foi utilizado para identificar o tipo de distribuição das variáveis contínuas. As variáveis numéricas contínuas foram expressas como mediana e intervalo interquartil (IIQ). Para variáveis contínuas, utilizamos o teste não-paramétrico de Kruskal-Wallis para comparar grupos com mais de duas categorias, e o teste U de Mann-Whitney para comparar grupos com duas categorias. O teste exato de Fisher foi utilizado para avaliar as associações entre as variáveis categóricas; valores de p menores que 0,05 foram considerados significativos. A análise estatística foi realizada com o software SPSS® para Windows®, versão 22.

## Resultados

**Figura 1:**
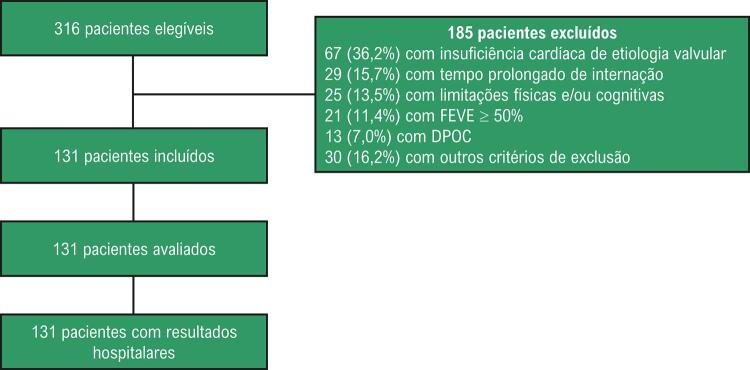
– Fluxo da seleção de pacientes. DPOC: doença pulmonar obstrutiva crônica; FEVE: fração de ejeção do ventrículo esquerdo.

Um total de 316 pacientes internados com ICD e elegíveis para o estudo foram avaliados. Deles, 185 indivíduos apresentavam pelo menos um critério de exclusão; 131 foram, por fim, incluídos no estudo e, depois, avaliados, como demonstrado na Figura 1 .

O tempo médio entre a internação e a avaliação nutricional foi de 6 (3-9) dias. A duração média da internação foi de 33 (20-57) dias. As características clínicas, demográficas e laboratoriais da amostra estão resumidas nas Tabelas 1 e 2, respectivamente.

**Tabela 1:** Características dos pacientes

Característica	N (%) / Mediana (IIQ)
Número de pacientes	131
Idade (anos)	56 (45-64)
Gênero masculino	85 (64,9)
Feminino	46 (35,1)
Etiologia da insuficiência cardíaca	
Cardiomiopatia dilatada	53 (40,5)
Doença de Chagas	42 (32,1)
Cardiopatia isquêmica	25 (19,1)
Hipertensão arterial	8 (6,1)
Outras etiologias	3 (2,3)
FEVE (%)	25 (20-30)
Diabetes Mellitus	37 (28,2)
Hipertensão arterial	56 (42,7)
Morte	34 (26)
Transplante cardíaco	38 (29)

*FEVE: fração de ejeção do ventrículo esquerdo; N: número de observações; IIQ: intervalo interquartil.*

**Tabela 2:** – Características clínicas e nutricionais de acordo com a etiologia da Doença de Chagas

	Total	Etiologia Doença de Chagas			Outras etiologias	Valor de p
	N (%) / Mediana (IIQ)	N (%) / Mediana (IIQ)			N (%) / Mediana (IIQ)	
Número de casos	131	42			89	
Idade (anos)	56 (45-64)	54 (43,8-65)			56 (45-63)	0,721
Gênero feminino [n (%)]	46 (35,1)	16 (38,1%)			30 (33,70)	0,69
FEVE(%)	25 (20-30)	25 (20-30)			25 (20,5-30)	0,438
BNP (pg/ml)	1093 (591-2149)	1424 (775,7-2945,7)			996 (495,5-2020)	0,022
Transplante ou morte [n (%)]	72 (55)	35 (83,3)			37 (41,6)	<0,001
IMC (kg/m^2^)	23,3 (20,6-26,7)	22,4 (19,9-25,3)			23,6 (20,8-27,3)	0,03
Categorização do IMC [n (%)]						
Baixo peso	33 (25,2)	11 (26,2)			22 (24,7)	0,169
Eutrofia	56 (42,7)	22 (52,4)			34 (38,2)	
Sobrepeso ou obesidade	42 (32,1)	9 (21,4)			33 (37,1)	
CB (cm)	28 (24-31,5)	27,1 (23,7-29,6)			28,5 (25-31,7)	0,042
DCT (mm)	16 (10-22,5)	15 (6,9-20,4)			16,5 (11,2-22,7)	0,1
AMB (cm^2^)	41,5 (34-49,3)	38,6 (33,1-45,2)			42,9 (36,4-50,3)	0,04
Baixa massa muscular [n (%)]	54 (41,2)	20 (47,6)			34 (38,2)	0,202
FPP (kg)	22,6 (16,1-30,3)	22,6 (15,3-30)			22,9 (16,3-30,7)	0,546
Baixa força muscular [n (%)]	65 (49,6)	24 (57,1)			41 (46,1)	0,16
Albumina (g/dl)	3,1 (2,8-3,5)	3,1 (2,8-3,4)			3,2 (2,7-3,6)	0,618
CTL (/mm^3^)	1380 (943-1893)	1254,5 (890,5-2037,7)			1386 (961-1851)	0,786
Colesterol Total (mg/dl)	133 (112,5-164)	140 (116,5-170,5)			132 (110,2-154,7)	0,317
Desnutrição [n (%)]	81 (61,8)	32 (76,2)			49 (55,1)	0,015
Caquexia [n (%)]	63 (48,1)	25 (59,5)			38 (42,7)	0,053

*FEVE: fração de ejeção do ventrículo esquerdo; BNP: peptídeo natriurético tipo-b; IMC: índice de massa corporal; DCT: dobra cutânea tricipital; CB: circunferência do braço; AMB: área muscular do braço; FPP: força de preensão palmar; CTL: contagem total de linfócitos; N: número de observações; IIQ: intervalo interquartil.*

Durante a internação, ocorreram 16 mortes (38,1%) em pacientes com a etiologia da Doença de Chagas, e em 18 (20,2%) pacientes com outras etiologias; o transplante cardíaco foi realizado em 19 (45,2%) pacientes com a etiologia da Doença de Chagas, e em 19 (21,3%) pacientes com outras etiologias (p<0,001).

### Características clínicas e nutricionais de acordo com a etiologia

As características clínicas e nutricionais de pacientes foram avaliadas de acordo com a etiologia da IC ( Tabela 2 ), e observamos que pacientes com a Doença de Chagas tinham IMC mais baixo (22,4 kg/m2 [19,9-25,3] vs. 23,6 kg/m2 [20,8-27,3], p=0,03), maior frequência de desnutrição de acordo com a ASG (76,2% vs. 55,1%, p=0,015) e maior média de BNP sérico (1,424 pg/mL [775,7-2,945,7] vs. 996 pg/mL [495,5 -2020], p = 0,022). Além disso, pacientes com Doença de Chagas apresentaram uma tendência a apresentar níveis mais baixos de hemoglobina (11,5 [9,9-13] g/dL vs. 12,4 [10,9-13,5] g/dL, p=0,053) ( Tabela 3 ).

**Tabela 3:** – Achados bioquímicos de acordo com a etiologia

	Total	Chagas	Outros	Valor de p
	N (%) / Mediana (IIQ)	N (%) / Mediana (IIQ)	N (%) / Mediana (IIQ)	
Número de casos	131	42	89	
Hemoglobina (g/dl)	12,1 (10,6-13,4)	11,5 (9,9-13)	12,4 (10,9-13,5)	0,053
Colesterol total (mg/dl)	133 (112,5-164)	140 (116,5-170,5)	132 (110,6-154,6)	0,317
HDL (mg/dl)	36 (27-47)	39 (29-48,5)	34 (26-47)	0,247
LDL (mg/dl)	82,5 (62,2-101)	85 (67-106)	81 (62-100)	0,233
Triglicérides (mg/dl)	78 (59,7-103)	74 (58,5-104)	80 (61,5-103)	0,944
Glicose (mg/dl)	105 (89-128)	104 (86-115)	106 (90-132)	0,322
Albumina (g/dl)	3,1 (2,8-3,5)	3,1 (2,8-3,4)	3,2 (2,8-3,6)	0,61
CTL (/mm^3^)	1380 (943-1893)	1254 (890-2037)	1386 (961-1851)	0,786
BNP (pg/ml)	1093 (591-2149)	1424 (775-2945)	996 (496-2020)	0,022
PCR (mg/l)	17,26 (7,53-32,91)	20,8 (7,8-39,8)	14,2 (6,5-29,1)	0,165
HbA_1_C (%)	6,2 (5,8-6,7)	6,1 (5,8-6,8)	6,2 (5,7-6,6)	0,621
Ureia (mg/dl)	65 (38-93)	62,5 (34,5-90,2)	65 (39,5-93,3)	0,871
Creatinina (mg/dl)	1,47 (1,14-2,01)	1,45 (1,2-1,9)	1,49 (1,12-2,09)	0,933

*IIQ: intervalo interquartil; HDL: lipoproteína de alta densidade; LDL: lipoproteína de baixa densidade; BNP: peptídeo natriurético tipo-b; CTL: contagem total de linfócitos; PCR: proteína C-reativa; HbA_1_C: hemoglobina glicada.*

### Variáveis nutricionais e resultados em pacientes com a etiologia da Doença de Chagas

As relações entre as variáveis nutricionais e clínicas e o desfecho hospitalar em pacientes com a etiologia da Doença de Chagas estão resumidos na Tabela 4 . Observou-se que a ocorrência de morte ou transplante cardíaco esteve associada à menos idade (67 [58-70] anos em pacientes que tiveram alta do hospital vs. 53 [41-60] anos em pacientes que morreram ou foram submetidos a um transplante cardíaco, p=0,007) e à desnutrição (3 [42,9%] pacientes que tiveram alta do hospital vs. 29 [82,9%] que morreram ou foram submetidos a um transplante cardíaco, p=0,007). A caquexia foi diagnosticada em apenas 2 (28,6%) pacientes que tiveram alta do hospital, e em 22 (62,9%) que morreram ou foram submetidos a um transplante cardíaco; porém, esta diferença não foi estatisticamente significativa (p=0,118).

**Tabela 4:** – Características clínicas e nutricionais e desfechos em pacientes com a etiologia da Doença de Chagas

	Alta	Morte/transplante	Valor de p
	N (%) / Mediana (IIQ)	N (%) / Mediana (IIQ)	
Número de casos	7	35	
Idade (anos)	67 (58-70)	53 (41-60)	0,007
Gênero feminino	4 (57,1)	12 (34,3)	0,397
FEVE (%)	30 (24-35)	25 (20-28)	0,085
BNP (pg/ml)	2497 (625-3773)	1365 (788-2673)	0,446
IMC (kg/m^2^)	22,9 (22,4-26,3)	22,1 (19,6-25,6)	0,122
Categorização do IMC			0,54
Baixo peso	3 (42,9)	8 (22,9)	
Eutrofia	3 (42,9)	19 (54,3)	
Sobrepeso/obesidade	1 (14,3)	8 (22,9)	
CB (cm)	27,5 (24,5-32,6)	27 (23,3-29,5)	0,466
DCT (mm)	20 (15-25,5)	13 (6,5-19)	0,193
AMB (cm^2^)	41,4 (32,7-51,1)	38,4 (33,3-45)	0,644
Baixa massa muscular	3 (42,9)	19 (54,3)	0,691
FPP (kg)	19 (11,9-24,3)	22,7 (16-30,7)	0,062
Baixa força muscular	5 (71,4)	19 (54,3)	0,679
Albumina (g/dl)	3 (2,8-3,4)	3,1 (2,8-3,4)	0,868
CTL (/mm^3^)	903 (612-1064)	1460 (1028-2069)	0,026
Colesterol total (mg/dl)	155,5 (129,8-182)	135 (113-173)	0,252
Desnutrição	3 (42,9)	29 (82,9)	0,043
Caquexia	2 (28,6)	22 (62,9)	0,118

*FEVE: fração de ejeção do ventrículo esquerdo; BNP: peptídeo natriurético tipo-b; IMC: índice de massa corporal; CB: circunferência do braço; DCT: dobra cutânea tricipital; AMB: área muscular do braço; FPP: força de preensão palmar; CTL: contagem total de linfócitos; N: número de observações; IIQ: intervalo interquartil.*

## Discussão

Considerando os resultados de forma conjunta, observam-se indícios de que há alta frequência de desnutrição e caquexia entre pacientes hospitalizados com ICD em nosso meio. Mais da metade da amostra (61,8%) apresentava algum grau de desnutrição, e quase metade (48,1%) foi diagnosticada com caquexia cardíaca. É importante mencionar que a presença de desnutrição esteve associada a maior risco de morte e transplante cardíaco durante a internação de pacientes com a cardiomiopatia chagásica.

Deve-se considerar que nossa amostra de pacientes tem características específicas em comparação a outros dados da literatura, principalmente idade média baixa, grande proporção de pacientes com a etiologia da Doença de Chagas e baixa proporção de pacientes com doença cardíaca isquêmica.^27^ Além disso, lidamos com uma população de alto risco, o que se reflete pelos marcadores da gravidade da doença (níveis mais baixos de FEVE e altos níveis de BNP circulante), assim como alta taxa de mortalidade e de transplantes. Esses achados podem ser explicados, dentre outros fatores, pelos critérios de seleção da amostra, que incluía indivíduos com FEVE menor que 50%, e as características do hospital onde o estudo foi realizado, que é um hospital terciário de referência.

Neste estudo, observamos a alta frequência de desnutrição diagnosticada pela ASG (61,8%). Um estudo espanhol também usou a ASG em pacientes internados com ICD e encontrou a desnutrição em 49,3% dos pacientes.^4^ Um estudo brasileiro com 53 pacientes com IC mostrou que a desnutrição estava presente, utilizando a ASG, em 51,9% dos pacientes.^28^ Outros métodos de avaliação foram utilizados e demonstraram taxas mais baixas de desnutrição, de 13 a 25,4%.^29 - 32^ Os dados apresentados aqui sugerem que a ASG pode ser ferramenta importante para o diagnóstico da desnutrição neste cenário.

Quando o IMC foi utilizado como indicador de status nutricional, a maioria da amostra se classificou como eutrófica (42,7%), seguida de sobrepeso ou obesidade (32,1%). Este achado contrasta com a alta porcentagem de desnutrição e caquexia. Acreditamos que este aspecto do estudo possa ser influenciado pelas limitações durante a coleta de dados em relação ao peso, já que uma medida direta do peso do paciente nem sempre foi clinicamente viável. Além disso, o peso pode se modificar com a retenção de líquidos no paciente com ICD, levando a um aumento não realista do IMC. Esta é provavelmente a explicação para a desnutrição na presença de sobrepeso ou obesidade medidos pelo IMC, uma ocorrência também reportada por outros autores.^31 , 33^ Além da influência da retenção de líquidos nesta amostra, uma redução significativa de massa muscular pode ter ocorrido, o que é um fenômeno conhecido como obesidade sarcopênica.^34^ Neste estudo, o diagnóstico de baixa massa muscular foi muito frequente (41,2%), assim como o diagnóstico de baixa força muscular (49,6%), o que aponta para certa perda de massa e função muscular nesta população. Nossos dados indicam que a classificação do IMC, como medida isolada, não é um bom indicador do status nutricional de pacientes com ICD.

Deve-se notar que a frequência de caquexia de 48,1% encontrada neste estudo difere-se das proporções achadas em outros estudos, que reportam prevalência de 10 a 16%.^1 , 35^ Porém, vale mencionar que foram utilizadas diferentes definições de caquexia; neste estudo, usamos os critérios propostos por Evans et al.^24^ Esses critérios consideram não só os aspectos como perda de peso não-intencional, mas também as variáveis bioquímicas. Além disso, essas amostras são muito diversas em termos da gravidade da doença cardíaca.

Nossos resultados indicam que pacientes com Doença de Chagas apresentam a doença mais grave, representada por níveis séricos mais altos de BNP e piores desfechos hospitalares. Os pacientes com Doença de Chagas também apresentaram pior status nutricional, representado por baixo peso corporal e IMC, pouca massa muscular e maior frequência de desnutrição na ASG. Possíveis mecanismos podem incluir maior envolvimento do lado direito do coração (manifestado pela ascite, hepatomegalia e edema no intestino) e maior atividade inflamatória em pacientes com Doença de Chagas.^36^

Acreditamos que o cenário clínico menos favorável, acompanhado pela maior gravidade da doença e atividade inflamatória do paciente com Doença de Chagas, possa ter um impacto na maior ocorrência de desnutrição nesta amostra, o que agrega ainda mais valor à avaliação nutricional, principalmente em relação à melhoria de resultados clínicos.

De acordo com esses achados, um estudo espanhol observou níveis mais altos de BNP circulante e maiores taxas de mortalidade entre pacientes desnutridos e hospitalizados com ICD, quando avaliados pela ASG.^4^ De acordo com esses mesmos autores, há sinais indicativos de uma relação bidirecional e mórbida entre a desnutrição e a insuficiência cardíaca. Nossos resultados reforçam esta teoria e indicam a importância de uma abordagem nutricional mais cuidadosa para a população com IC e Doença de Chagas.

Também observamos que a população mais nova e a presença de desnutrição estiveram associadas à maior ocorrência de mortes e transplante cardíaco em pacientes com a etiologia da Doença de Chagas. Embora a caquexia tenha sido duas vezes mais comum em pacientes que morreram ou foram submetidos a um transplante cardíaco durante a internação, ao compará-los com pacientes que tiveram alta, a diferença não foi estatisticamente diferente devido ao número limitado de pacientes em nossa amostra. A idade como fator protetivo pode ser explicado pelo fato de que a idade é um fator limitante para indicação do procedimento de transplante. A maior frequência de mortes dentre pacientes desnutridos e hospitalizados com ICD, e avaliados pela ASG, já foi anteriormente descrita na literatura.^4^ No presente estudo, pôde-se observar que esta relação se mantém ao avaliar pacientes com ICD de etiologia da Doença de Chagas, o que reforça o status nutricional como importante aspecto que influencia o desfecho de pacientes com IC. Além disso, alguns autores buscaram registrar a associação entre o status nutricional e o prognóstico de pacientes com IC, e esses estudos mostram que a desnutrição, diagnosticada por diferentes métodos, apareceu como um fator de risco independente para mortalidade por todas as causas.^4 , 29 , 30 , 31 , 33^

Este estudo apresenta limitações que devem ser reconhecidas. Por ser um ensaio clínico não-randomizado, não foi possível descartar a presença de variáveis de confusão ao comparar grupos de pacientes. A grande proporção de pacientes com Doença de Chagas e a alta gravidade dos casos impedem a extrapolação de nossos achados para outros cenários clínicos. Também, este estudo não analisou a influência das variáveis clínicas indicando congestão, como edema periférico, hepatomegalia e ascite. Assim, não podemos excluir a possibilidade de viés nas medidas de peso corporal total.

## Conclusões

Ao todo, nossos resultados indicam que pacientes com Doença de Chagas internados com ICD costumam apresentar distúrbios nutricionais, principalmente desnutrição; é importante mencionar que este achado esteve associado à ocorrência de morte e transplante cardíaco durante a internação.
