# Supplementary material for: Desnutrição e Caquexia na Insuficiência Cardíaca Descompensada e Cardiomiopatia Chagásica: Ocorrência e Associação com Desfechos Hospitalares
Source: Arq Bras Cardiol. 2021 Nov 17;118(1):3–11. [Article in Portuguese] doi: 10.36660/abc.20200644 (PMC8959049; doi:10.36660/abc.20200644)
Supplement: Translation

# Translation: Undernutrition and Cachexia in Patients with Decompensated Heart Failure and Chagas Cardiomyopathy: Occurrence and Association with Hospital Outcomes

**Authors:** Larissa Candido Alves Tavares, Silvia Helena Gelás Lage, Edimar Alcides Bocchi, Victor Sarli Issa

**Affiliations:** 1 Brasil Universidade de São Paulo Instituto do Coração - Serviço de Nutrição e Dietética, São Paulo, SP – Brasil; 2 Brasil Universidade de São Paulo Instituto do Coração - Unidade de Terapia Intensiva Clínica, São Paulo, SP – Brasil; 3 Brasil Universidade de São Paulo Instituto do Coração - Insuficiência Cardíaca, São Paulo, SP – Brasil

**Keywords:** Chagas Cardiomyopathy, Heart Failure, Malnutrition, Cachexia, Nutrition Assessment

## Abstract

**Background:**

Nutritional disorders are common among patients with heart failure (HF) and associated with poor prognosis. Importantly, some populations of patients, like the ones with Chagas disease, are frequently excluded from most analyses.

**Objective:**

We sought to study the occurrence of undernutrition and cachexia in patients with Chagas disease during episodes of decompensated HF (DHF) as compared to other etiologies, and to investigate the influence of these findings on hospital outcomes.

**Methods:**

We performed a consecutive case series study with patients hospitalized with DHF. Patients underwent the Subjective Global Assessment of nutritional status (SGA), besides anthropometric and laboratorial measures, and were evaluated for the occurrence of cachexia, low muscle mass and strength. We studied the occurrence of death or urgent heart transplantation during hospitalization.

**Results:**

Altogether, 131 patients were analyzed and 42 (32.1%) had Chagas disease. Patients with Chagas disease had lower Body Mass Index (BMI) (22.4 kg/m^2^[19.9-25.3] vs. 23.6 kg/m2 [20.8-27.3], p=0.03), higher frequency of undernutrition (76.2% vs 55.1%, p=0.015) and higher occurrence of death or transplant (83.3% vs. 41.6%, p<0.001). We found that, in patients with Chagas etiology, the occurrence of death or cardiac transplantation were associated with undernutrition (3 [42.9%] patients with hospital discharge vs 29 [82.9%] patients with death or heart transplant, p=0.043).

**Conclusions:**

Taken together, our results indicate that patients with Chagas disease hospitalized with DHF often present with nutritional disorders, especially undernutrition; importantly, this finding was associated with the occurrence of death and heart transplant during hospitalization.

## Introduction

Nutritional disorders are one of the main clinical manifestations in patients with heart failure (HF), and are the result of systemic derangements involving metabolic, endocrine and inflammatory pathways, as well as organ dysfunction. The occurrence of nutritional disorders in the setting of chronic HF has been consistently associated to reduction in the quality of life and limited survival of patients.^1 - 5^

Nutritional disorders may be manifested in various clinical forms, which is reflected in an extensive and heterogeneous terminology.^1 - 5^ More recently, a standardization of terms was proposed^6 , 7^ and suggested that *undernutrition* might be defined as a condition in which the energy and protein reserves of the organism are depleted, whereas *cachexia* was characterized by the intense loss of adipose and muscle mass accompanied by the increased inflammation and protein catabolism due to chronic diseases.

It is estimated that the frequency of cachexia and undernutrition associated with HF ranges according to the studied population and the diagnostic criteria. In addition, frequent water retention in this population makes basic measures for nutritional assessment, such as weight and body mass index (BMI), a challenge in clinical practice. The estimation is that about 15% of patients with chronic HF are cachectic, and up to 50% of patients may show signs of undernutrition;^2^ additionally, a prevalence of 19.5% of muscular atrophy has been reported.^3^ However, most of the data derives from patients with chronic HF, and few studies have been systematically reported considering the nutritional evaluation of patients during episodes of acute decompensation. In one of the few studies about decompensated heart failure (DHF), a 41.9% prevalence of moderate undernutrition was reported, whereas 7.4% was demonstrated for severe undernutrition, as evaluated by the Subjective Global Assessment of nutritional status (SGA).^4^ Although the SGA was described 20 years ago,^8^ the method showed a good diagnostic precision when performed by trained observers, besides being simple, inexpensive and noninvasive.^9^ The SGA assesses the nutritional status diagnosis, unlike other tools, such as the Prognostic Nutritional Index (PNI) and the Controlling Nutritional Status (CONUT) score. Even though they are frequently used in clinical practice, they are fundamentally screening tools and do not apply for the diagnosis of undernutrition.^10^

Nutritional data are even more scarce in other clinical scenarios, such as in patient populations frequently excluded from clinical trials and prospective studies, often referred to as neglected conditions,^11^ which is the case for Chagas heart disease.^12^ Despite the evidence indicating that patients with Chagas heart disease have worse prognosis in cases of both chronic^13^ and decompensated HF^14^ when compared to other etiologies, the extent to which the nutritional characteristics of Chagas patients differ and their impact on outcomes remain largely unknown.

Therefore, we hypothesized that nutritional disorders may be common in patients with DHF and have an influence on prognosis; we further hypothesized that the nutritional characteristics of patients may differ in terms of intensity, clinical presentation and prognostic implication according to the etiology of HF.

The aim of this study was to evaluate the occurrence of malnutrition and cachexia in patients with DHF and to observe clinical and nutritional characteristics and their influence on in-hospital outcomes according to the presence of Chagas etiology.

## Patients and Methods

### Study Design

We performed a consecutive case series study with hospitalized patients diagnosed with DHF at the Heart Institute ( *InCor* ) - Hospital das Clínicas, Medical School of Universidade de São Paulo, a tertiary academic hospital specialized in cardiac diseases. The study protocol was approved by the Research Ethics Committee of Hospital das Clínicas, Medical School of Universidade de São Paulo, and all patients signed the informed consent form. The analyses and patients were followed up until hospital discharge.

Patients aged 18 years or older, hospitalized with DHF and with systolic left ventricular dysfunction, were considered eligible for the study. Left ventricular ejection fraction (LVEF) lower than 50%, measured by transthoracic echocardiography, was considered as an indication of systolic dysfunction. The tests performed up to twelve months before the episode of decompensation were considered valid. The first inclusion occurred in February, 2016, and the last inclusion was in April, 2018.

The identification of cases was based on medical records that registered the diagnosis of DHF. In order to be included in the study, patients needed to meet the modified Framingham criteria for the diagnosis of HF.^15^ The following criteria were considered as indications of decompensation: the presence of any new symptom or aggravation of current ones (shortness of breath, orthopnea, peripheral edema, ascites), combined with any sign of congestion or hypoperfusion (tachycardia, hypotension, dyspnea, tachypnea, lower limb edema, pulmonary crepitations, pleural effusion, ascites, hepatomegaly, increased central venous pressure and presence of hepatojugular reflux). The diagnosis of a Chagas etiology was based on positive serology for Chagas disease, along with typical clinical presentation and exclusion of other etiologies.

Exclusion criteria were: presence of congenital heart disease; restrictive heart disease; testing positive for HIV; active alcoholism; life-limiting chronic obstructive pulmonary disease (COPD); presence of Chagas megacolon and / or megaesophagus; continuous use of corticosteroids or immunosuppressants in the past 3 months; malignant neoplasm; pulmonary embolism in the past 6 months; major surgeries or serious infections in the past 30 days; primary valvular diseases as cause of HF; physical limitations that prevented anthropometric measurements in a minimally proper manner; pregnancy; impossibility of performing clinical and nutritional anamnesis with patient, family member or partner.

From July 2017 onwards, the time greater than 7 days of hospitalization before inclusion in the study was added as an exclusion criterion.

### Studied outcomes

Patients were followed up from hospital admission to hospital discharge, in cases of death or urgent heart transplant.

### Clinical and nutritional variables

The clinical variables of the sample were obtained from the review of the medical evolution registered in electronic medical records and interviews with patients and / or family members.

Values of hemoglobin (Hb), glucose, glycosylated hemoglobin (HbA1C), total cholesterol, LDL cholesterol, HDL cholesterol, triglycerides (TG), albumin, total lymphocyte count (TLC), C-reactive protein (CRP) were collected at hospital admission. For the B-type natriuretic peptide (BNP), we considered any measure taken up to 6 months before hospital admission as valid.

The non-biochemical variables related to the nutritional status were obtained from an interview with the patient and/or accompanying person, and the assessment of anthropometric measures was performed by a single clinical nutritionist. The measures of height, current and usual weight were referred by the patient. The weight and height of patients who were able to walk were also verified using a digital scale with a coupled stadiometer, with capacity of up to 150kg and 190cm (Filizola®). In cases in which individuals had edema at the time of assessment, the current edema-free weight was reported by the patient, and the individual was not weighed on the scale.

Patients who could not walk because of medication, but could stand up, were weighed with a portable digital scale (EKS 8873 DOMUS Plataforma ABS®). The height of these same patients was measured using a portable stadiometer (Wood Portátil Compact®). Individuals who were unable to report weight and height and those who were bed-restricted, and, therefore, could not be subjected to measuring, had their heights and weights estimated by predictive formulas. These formulas consider knee height, age, arm circumference (AC) and ethnicity.^16 - 18^

AC was measured using an inelastic tape measure at the midpoint of the arm (between the acromion and the olecranon process), with the arm stretched freely along the body. The triciptal skinfold (TS) was measured using a skinfold caliper (Sahean®). The fold was pinched with the fingers at the midpoint of the arm (between the acromion and the olecranon process), with the arm stretched freely along the body. The measure was repeated three times, and the mean of measures was used for analysis. Hand grip strength (HGS), considered as the measure of isometric force, of the handshake in the dominant upper limb, was performed with a digital hand held dynamometer (MG-4800®). The test was performed with the individual sitting down or with the head elevated at least 30º when the patient was using an intra-aortic balloon, and the arm forming a 90º angle with the supported elbow. Three measurements of maximum force were performed with 10-second intervals between each execution, and the average of the three values was considered.

### Diagnosis and categorization

The BMI was categorized according to the World Health Organization (WHO)^19^ criteria for adults, and according to Pan American Health Organization (PAHO)^20^ for the elderly aged more than sixty years. For the purposes of undernutrition diagnosis, the SGA was used.^8^ The application of all questionnaires and physical examination was performed by a single clinical nutritionist. The identification of individuals with low muscle mass was performed by calculating the arm muscle area (AMA), obtained from arm circumference (AC) and TS, using the following formula:


$$\text{ AMA }\left({cm}^{2}\right)={\left[AC(cm)-\left(TS(cm)\times \prod ÷10\right)\right]}^{2}/4\Pi$$

Individuals with low muscle mass were those who were below the 10th percentile when compared to a reference population. The comparison values according to percentile ranges of the reference population were obtained from the distribution presented by Frisancho^21^ for adults, and the distribution presented by Burr and Phillips^22^ for the elderly.

The diagnosis of reduced muscle strength was performed using HGS. For purposes of analysis, individuals with low muscular strength were considered those who presented values equal to or lower than those considered low by Mathiowetz et al.^23^ in a healthy population according to gender and age group.

The diagnosis of cardiac cachexia was performed based on the criteria proposed by Evans *et al* .^24^ This definition involves the presence of a chronic disease diagnosis (a criterion filled in this study by the presence of HF), associated with a 5% weight loss in relation to the usual weight in a maximum period of 12 months, or BMI of less than 20kg/m^2^ , and accompanied by at least three of the following criteria: fatigue, anorexia, low muscle strength, low muscle mass and biochemical abnormalities, such as increased inflammatory markers, anemia, and low serum albumin.

The severity of HF was estimated in the present study by observing the occurrence of higher levels of circulating BNP and lower values of LVEF, as shown in the literature.^25 , 26^

### Statistical Analysis

The Kolmogorov-Smirnov test was used to identify the type of distribution of the continuous variables. Continuous numerical variables were expressed as median and interquartile range (IQR). For continuous variables, the non-parametric Kruskal-Wallis tests were used to compare groups with more than two categories, and the Mann-Whitney U test to compare groups with two categories. The Fisher’s exact test was used to analyze the associations between categorical variables; P values lower than 0.05 were considered significant. Statistical analysis was performed using the SPSS® software for Windows®, version 22.

## Results

A total of 316 hospitalized patients admitted with DHF and eligible for the study were evaluated. Of these, 185 individuals met at least one exclusion criteria and 131 were eventually included in the study and further evaluated, as shown in Figure 1 .

**Figure 1:** – Patient selection flowchart. COPD: chronic obstructive pulmonary disease; LVEF: left ventricular ejection fraction.

The median time between hospitalization and the nutritional evaluation was 6 (3-9) days. The median duration of hospital stay was 33 (20-57) days. The clinical, demographic and laboratory characteristics of the sample are summarized in Tables 1 and 2, respectively.

**Table 1:** – Patients’ characteristics

Characteristic	N (%) / Median (IQR)
Number of patients	131
Age (years)	56 (45-64)
Gender male	85 (64.9)
Female	46 (35.1)
Heart failure etiology	
Dilated cardiomyopathy	53 (40.5)
Chagas disease	42 (32.1)
Ischemic cardiomyopathy	25 (19.1)
Arterial hypertension	8 (6.1)
Other etiologies	3 (2.3)
LVEF (%)	25 (20-30)
Diabetes Mellitus	37 (28.2)
Arterial hypertension	56 (42.7)
Death	34 (26)
Heart transplant	38 (29)

*LVEF: left ventricular ejection fraction; N: number of observations; IQR: interquartile range.*

**Table 2:** – Clinical and nutritional characteristics according to Chagas etiology

	Total	Chagas etiology	Other etiologies	p-value
	N (%) / Median (IQR)	N (%) / Median (IQR)	N (%) / Median (IQR)	
Number of cases	131	42	89	
Age (years)	56 (45-64)	54 (43.8-65)	56 (45-63)	0.721
Female gender [n (%)]	46 (35.1)	16 (38.1%)	30 (33.70)	0.69
LVEF(%)	25 (20-30)	25 (20-30)	25 (20.5-30)	0.438
BNP (pg/ml)	1093 (591-2149)	1424 (775.7-2945.7)	996 (495.5-2020)	0.022
Transplant or death [n (%)]	72 (55)	35 (83.3)	37 (41.6)	<0.001
BMI (kg/m^2^)	23.3 (20.6-26.7)	22.4 (19.9-25.3)	23.6 (20.8-27.3)	0.03
BMI categorization [n (%)]				
Underweight	33 (25.2)	11 (26.2)	22 (24.7)	0.169
Eutrophy	56 (42.7)	22 (52.4)	34 (38.2)	
Overweight or obesity	42 (32.1)	9 (21.4)	33 (37.1)	
AC (cm)	28 (24-31.5)	27.1 (23.7-29.6)	28.5 (25-31.7)	0.042
TS (mm)	16 (10-22.5)	15 (6.9-20.4)	16.5 (11.2-22.7)	0.1
AMA (cm^2^)	41.5 (34-49.3)	38.6 (33.1-45.2)	42.9 (36.4-50.3)	0.04
Low muscle mass [n (%)]	54 (41.2)	20 (47.6)	34 (38.2)	0.202
HGS (kg)	22.6 (16.1-30.3)	22.6 (15.3-30)	22.9 (16.3-30.7)	0.546
Low muscle strength [n (%)]	65 (49.6)	24 (57.1)	41 (46.1)	0.16
Albumin (g/dl)	3.1 (2.8-3.5)	3.1 (2.8-3.4)	3.2 (2.7-3.6)	0.618
TLC (/mm^3^)	1380 (943-1893)	1254.5 (890.5-2037.7)	1386 (961-1851)	0.786
Total Cholesterol (mg/dl)	133 (112.5-164)	140 (116.5-170.5)	132 (110.2-154.7)	0.317
Undernutrition [n (%)]	81 (61.8)	32 (76.2)	49 (55.1)	0.015
Cachexia [n (%)]	63 (48.1)	25 (59.5)	38 (42.7)	0.053

*LVEF: left ventricular ejection fraction; BNP: B-type natriuretic peptide; BMI: body mass index; TS: tricipital skinfold; AC: arm circumference; AMA: arm muscle area; HGS: hand grip strength; TLC: total lymphocyte count; N: number of observations; IQR: interquartile range.*

During hospital admission, death occurred in 16 (38.1%) patients with Chagas etiology and in 18 (20.2%) patients with other etiologies; heart transplant was performed in 19 (45.2%) patients with Chagas etiology, and in 19 (21.3%) patients with other etiologies (p<0.001).

### Clinical and nutritional characteristics according to etiology

Clinical and nutritional characteristics of patients were analyzed according to the etiology of HF ( Table 2 ), and we observed that patients with Chagas disease had lower BMI (22.4 kg/m2 [19.9-25.3] vs 23.6 kg/m2 [20.8-27.3], p = 0.03), higher frequency of undernutrition by SGA (76.2% vs 55.1%, p = 0.015), higher median serum BNP at admission (1,424 pg/mL [775.7-2,945.7] vs 996 pg/mL [495.5 -2020], p = 0.022). Additionally, Chagas patients had a tendency towards lower levels of hemoglobin (11.5 [9.9-13] g/dL vs 12.4 [10.9-13.5] g/dL, p=0.053) ( Table 3 ).

**Table 3:** – Biochemical findings according to etiology

	Total	Chagas	Others		p-value
	N (%) / Median (IQR)	N (%) / Median (IQR)	N (%) / Median (IQR)		
Number of cases	131	42	89		
Hemoglobin (g/dl)	12.1 (10.6-13.4)	11.5 (9.9-13)	12.4 (10.9-13.5)		0.053
Total Cholesterol (mg/dl)	133 (112.5-164)	140 (116.5-170.5)	132 (110.6-154.6)		0.317
HDL (mg/dl)	36 (27-47)	39 (29-48.5)	34 (26-47)		0.247
LDL (mg/dl)	82.5 (62.2-101)	85 (67-106)	81 (62-100)		0.233
Triglycerides (mg/dl)	78 (59.7-103)	74 (58.5-104)	80 (61.5-103)		0.944
Glucose (mg/dl)	105 (89-128)	104 (86-115)	106 (90-132)		0.322
Albumin (g/dl)	3.1 (2.8-3.5)	3.1 (2.8-3.4)	3.2 (2.8-3.6)		0.61
TLC (/mm^3^)	1380 (943-1893)	1254 (890-2037)	1386 (961-1851)		0.786
BNP (pg/ml)	1093 (591-2149)	1424 (775-2945)	996 (496-2020)		0.022
CRP (mg/l)	17.26 (7.53-32.91)	20.8 (7.8-39.8)	14.2 (6.5-29.1)		0.165
HbA_1_C (%)	6.2 (5.8-6.7)	6.1 (5.8-6.8)	6.2 (5.7-6.6)		0.621
Urea (mg/dl)	65 (38-93)	62.5 (34.5-90.2)	65 (39.5-93.3)		0.871
Creatinine (mg/dl)	1.47 (1.14-2.01)	1.45 (1.2-1.9)	1.49 (1.12-2.09)		0.933

*IQR: interquartile range; HDL: high density lipoprotein; LDL: low density lipoprotein; BNP: B-type natriuretic peptide; TLC: total lymphocyte count; CRP: C-reactive protein; HbA_1_C: glycosylated hemoglobin.*

### Nutritional variables and outcome in patients with Chagas etiology

The relationships between nutritional and clinical variables and hospital outcome in patients with the Chagas etiology are summarized in Table 4 . We found that the occurrence of death or heart transplant were associated with younger age (67 [58-70] years of age in patients who were discharged from the hospital vs 53 [41-60] years in patients who died or had a heart transplant, p=0.007) and undernutrition (3 [42.9%] patients who were discharged from the hospital vs 29 [82.9%] patients who died or had a heart transplant, p=0.007). Cachexia was found in only 2 (28.6%) patients who were discharged from the hospital, and in 22 (62.9%) who died or had a heart transplant; however, this difference was not statistically significant (p=0.118).

**Table 4:** – Clinical and nutritional characteristics and outcomes in patients with Chagas etiology

	Discharge	Death/transplant	p-value
	N (%) / Median (IQR)	N (%) / Median (IQR)	
Number of cases	7	35	
Age (years)	67 (58-70)	53 (41-60)	0.007
Female gender	4 (57.1)	12 (34.3)	0.397
LVEF (%)	30 (24-35)	25 (20-28)	0.085
BNP (pg/ml)	2497 (625-3773)	1365 (788-2673)	0.446
BMI (kg/m^2^)	22.9 (22.4-26.3)	22.1 (19.6-25.6)	0.122
BMI categorization			0.54
Underweight	3 (42.9)	8 (22.9)	
Eutrophy	3 (42.9)	19 (54.3)	
Overweight/obesity	1 (14.3)	8 (22.9)	
AC (cm)	27.5 (24.5-32.6)	27 (23.3-29.5)	0.466
TS (mm)	20 (15-25.5)	13 (6.5-19)	0.193
AMA (cm^2^)	41.4 (32.7-51.1)	38.4 (33.3-45)	0.644
Low muscle mass	3 (42.9)	19 (54.3)	0.691
HGS (kg)	19 (11.9-24.3)	22.7 (16-30.7)	0.062
Low muscle strengh	5 (71.4)	19 (54.3)	0.679
Albumin (g/dl)	3 (2.8-3.4)	3.1 (2.8-3.4)	0.868
TLC (/mm^3^)	903 (612-1064)	1460 (1028-2069)	0.026
Total Cholesterol (mg/dl)	155.5 (129.8-182)	135 (113-173)	0.252
Undernutrition	3 (42.9)	29 (82.9)	0.043
Cachexia	2 (28.6)	22 (62.9)	0.118

*LVEF: left ventricular ejection fraction; BNP: B-type natriuretic peptide; BMI: body mass index; AC: arm circumference; TS: tricipital skinfold; AMA: arm muscle area; HGS: hand grip strength; TLC: total lymphocytes count; N: number of observations; IQR: interquartile range.*

## Discussion

Considered together, our results indicate there is a high frequency of undernutrition and cachexia among hospitalized patients with DHF in our setting. More than half of the sample (61.8%) had some level of undernutrition, and almost half (48.1%) received a diagnosis of cardiac cachexia. Importantly, the presence of undernutrition was associated with increased risk of death and heart transplant during admission in patients with Chagas cardiomyopathy.

It should be acknowledged that our patient sample has specific characteristics as compared to other data from the literature, specially low mean age, large proportion of patients with Chagas etiology, and low proportion of patients with ischemic heart disease.^27^ Additionally, we dealt with a high risk population, which is reflected by markers of disease severity (reduced LVEF and high levels of circulating BNP), as well high mortality and transplant rates. These findings can be explained, among other factors, by the selection criteria of the sample, which included individuals with LVEF lower than 50%, and the characteristics of the hospital where the study was performed, which was a tertiary referral hospital.

In the present study, we could observe high frequency of undernutrition as diagnosed by SGA in our setting (61.8%). A Spanish study also used SGA in hospitalized DHF patients and found undernutrition in 49.3% of the patients.^4^ A Brazilian study with 53 patients with HF showed presence of undernutrition using the SGA in 51.9% of the patients.^28^ Other methods of evaluation were tested and found lower rates of undernutrition, ranging from 13% to 25.4%.^29 - 32^ The present data suggest that SGA may be a useful tool for the diagnosis of undernutrition in this setting.

When BMI was used as an indicator of nutritional status, we found most of the sample classified as eutrophic (42.7%), followed by overweight or obesity (32.1%). This finding is in contrast with the high percentage of undernutrition and cachexia. We believe that this aspect of the study may be influenced by the limitations during data collection regarding the weight of the present study, since a direct measure of the patient weight was not always clinically feasible. In addition, the measured weight may be modified by the fluid retention in the patient with DHF, leading to an unrealistic increase of the BMI. This is probably the explanation for undernutrition, in the presence of overweight or obesity as measured by BMI, an occurrence also reported by other authors.^31 , 33^ In addition to the influence of fluid retention in this sample, a significant reduction of muscle mass may have occurred, which is a phenomenon known as sarcopenic obesity.^34^ In the present study, the diagnosis of low muscle content was very frequent (41.2%), as well as the diagnosis of low muscle strength (49.6%), which points to a certain loss of muscle mass and function in this population. Our data indicate that BMI classification, as an isolated measure, is not a good indicator of the nutritional status of patients with DHF.

It must be noted that the frequency of cachexia of 48.1% found in this study differs from the proportions found in other studies, which report a prevalence from 10 to 16%.^1 , 35^ However, it is worth mentioning that different definitions of cachexia were used, and in the present study the criteria proposed by Evans et al.^24^ were adopted; these criteria take into consideration not only aspects such as unintentional weight loss, but also biochemical variables. In addition, these are very diverse samples in terms heart disease severity.

Our results indicate that patients with Chagas disease present with more severe disease, as represented by higher serum levels of BNP and worse hospital outcomes. Chagas disease patients also had worse nutritional status, represented by lower body weight and BMI, lower muscle mass and higher frequency of undernutrition by SGA. Possible mechanisms may involve increased right heart side involvement (as manifested by ascites, hepatomegaly and intestinal loop edema) and increased inflammatory activity in patients with Chagas disease.^36^

We believe that the less favorable clinical picture, accompanied by the greater severity of the disease and the inflammatory activity of the patient with Chagas disease, may have an impact on the greater occurrence of undernutrition in this sample, which adds even more value to nutritional assessment, especially regarding the improvement of clinical outcomes.

In accordance with these findings, a Spanish study found higher levels of circulating BNP and higher death rates among malnourished patients with DHF hospitalized and evaluated by the SGA.^4^ According to these same authors, there are indicative signs of a bidirectional and morbid relationship between undernutrition and heart failure. Our results reinforce this theory and indicate the importance of a more careful nutritional approach for the population with HF of Chagas disease.

We found that younger age and the presence of undernutrition were associated with the higher occurrence of death and heart transplant in patients with Chagas etiology. Even though cachexia was twice as frequent in patients who died or had a heart transplant during hospital stay, when comparing them to patients who were discharged from the hospital the difference was not statistically different due to the limited number of patients in our sample. The age as a protective factor can be explained by the fact that age is a limiting factor for indicating a heart transplant procedure. The higher frequency of death among malnourished patients hospitalized with DHF and evaluated by the SGA had been previously described in the literature.^4^ In this study, we could see that this relationship remains when evaluating patients with DHF of Chagas etiology, which reinforces the nutritional status as an important aspect for the outcome of patients with HF. In addition, some authors have sought to register the association between nutritional status and prognosis in patients with HF, and these studies show that undernutrition, diagnosed by different methods, appeared as an independent risk factor for all-cause mortality.^4 , 29 , 30 , 31 , 33^

The present study presents limitations which should be acknowledged. As a non-randomized clinical study, it was not possible to rule out the presence of confounding variables when comparing patient groups. The large proportion of patients with Chagas disease and the high severity cases of the patients hinder the extrapolation of our findings to other clinical scenarios. Furthermore, this study did not analyze the influence of clinical variables indication congestion, such as peripheral edema, hepatomegaly and ascites. Therefore, we cannot exclude the possibility of bias in the measurement of total body weight.

## Conclusions

Taken together, our results indicate that patients with Chagas disease hospitalized with DHF often present with nutritional disorders, especially undernutrition; importantly, this finding was associated with the occurrence of death and heart transplant during hospitalization.
